# Early p53 mutations in nondysplastic Barrett's tissue detected by the restriction site mutation (RSM) methodology

**DOI:** 10.1038/sj.bjc.6600891

**Published:** 2003-04-15

**Authors:** G J S Jenkins, S H Doak, A P Griffiths, N Tofazzal, V Shah, J N Baxter, J M Parry

**Affiliations:** 1 Swansea Clinical School, University of Wales Swansea, Singleton Park, Swansea SA28PP, UK; 2 School of Biological Sciences, University of Wales Swansea, Singleton Park, Swansea SA28PP, UK; 3 Department of Histopathology, Morriston Hospital, Morriston, Swansea SA6 6NL, UK; 4 Department of Histopathology, Singleton Hospital, Swansea SA2 8QA, UK; 5 Department of Surgery, Morriston Hospital, Morriston, Swansea SA6 6NL, UK

**Keywords:** mutation, p53 gene, biomarker, Barrett's oesophagus

## Abstract

Barrett's oesophagus is a premalignant condition whose incidence is rising dramatically. Molecular markers are urgently needed to identify Barrett's patients at the highest risk of cancer progression. To this end, we have used a rapid molecular technique, restriction site mutation (RSM), to detect low-frequency mutations in the p53 tumour suppressor gene in premalignant Barrett's tissues of cancer-free patients. In total, 38 endoscopically diagnosed Barrett's patients with a range of histological stages of Barrett's progression, plus four control patients without Barrett's oesophagus, were analysed for early p53 mutations. Tissue samples taken from these patients (93 samples in total) were analysed for the presence of low-frequency p53 mutations at hotspot codons: 175, 213, 248, 249, 282. In total, 13 of the 38 Barrett's patients were shown to possess a p53 mutation in at least one sample (no mutations in the four control patients). Although no statistically significant associations were found, p53 mutations reflected histological progression in Barrett's patients with p53 mutations found in 30% of metaplasia patients (*P*=0.4) and low-grade dysplasia patients (*P*=0.33) and 45% of high-grade dysplasia patients (*P*=0.15). Detected p53 mutations were mainly GC to AT transitions at CpG sites.

Barrett's metaplasia is a precursor of adenocarcinoma of the oesophagus, the incidence of which is rising dramatically in Western countries (Watson, 2000). Metaplastic Barrett's epithelium may progress through low-grade dysplasia (LGD) and high-grade dysplasia (HGD) to cancer. Patients with a metaplastic Barrett's segment have a 30–125-fold greater risk of adenocarcinoma than non-Barrett's patients (Cameron *et al*, 1985). The driving force behind cancer development in Barrett's tissues is thought to be oesophageal inflammation induced by chronic reflux of stomach acid and duodenal fluid (Chen and Yang, 2001). Inflamed tissues are known to be cancer prone (Ambs *et al*, 1999), partly because of their increased cell proliferation rates (Sjoqvist *et al*, 1999). The reactive oxygen species (ROS) produced during such inflammation (e.g. superoxide, hydrogen peroxide, nitric oxide) are complete carcinogens in their own right (Cerutti and Trump, 1991; Dreher and Junod, 1996). However, coincident exposure of inflamed tissues to mutagens (possibly bile acids in the case of Barrett's patients) can exacerbate carcinogenesis.

Progression of Barrett's metaplasia to adenocarcinoma is accompanied by a range of genetic alterations (Fitzgerald and Triadafilopoulos, 1998; Jankowski *et al*, 1999; Jenkins *et al*, 2002), including loss of p53 tumour suppressor function. The most common mechanism of p53 loss is through the accumulation of mutations at one of eight hotspot codons (Hainaut and Hollstein, 2000). p53 loss is significant in cancer development because of the role that p53 plays in maintaining genomic stability. It is well known that p53 loss leads to increased spontaneous mutation rates (Havre *et al*, 1995), chromosome instability (Bouffler *et al*, 1995) and aneuploidy (Fukasawa *et al*, 1996). These latter two events are characteristic of Barrett's progression (Jenkins *et al*, 2002). Indeed, it has been shown that p53 mutation precedes (and perhaps leads to) aneuploidy in Barrett's tissues (Neshat *et al*, 1994; Prevo *et al*, 1999).

The role of p53 mutation in Barrett's tumour progression has been studied previously, with p53 mutations being detected in Barrett's tumours and in adjacent dysplastic tissue. However, p53 mutation has been poorly studied in premalignant Barrett's tissues, especially in cancer-free patients (Prevo *et al*, 1999). This lack of p53 mutation data has led to controversy with respect to the timing of p53 mutation in Barrett's progression (Fitzgerald and Triadafilopoulos, 1998). Table 1

**Table 1:** Review of published molecular data on p53 mutations in Barrett's tissues

**Tissue**	**% mutated**	**Codons**	**Mutation**	**Method**	**Reference**	**Notes**
Tumour	50% (5/10)	176, 245, 248, 282, 286	GC to AT, GC to TA	DGGE and sequencing	Audrezet *et al* (1996)	One case surrounding HGD tissue also mutated
Tumour	7% (1/14)	273	GC to AT	SSCP and sequencing	Casson *et al* (1991)	Four out of seven surrounding metaplasia and LGD tissue contained mutation
Tumour	50% (30/59)	173-175, 248, 273, 282	GC to AT	SSCP and sequencing	Schneider *et al* (2000)	p53 mutation prognostically significant
Tumour	88% (15/17)	175, 196, 213, 245	GC to AT	DGGE and sequencing	Hamelin *et al* (1994)	Mutation in surrounding HGD tissue but not metaplasia
Tumour	46% (23/50)	248, 275, 278	GC to AT	SSCP and sequencing	Schneider *et al* (1996)	Four cases adjacent mucosa (dysplastic) contained mutation
Tumour	57% (17/30)	175, 248, 249	GC to AT	SSCP and sequencing	Bian *et al* (2001)	Mutations in HGD and LGD, but not metaplasia
Tumour/HGD	64% (9/14)	213, 248, 273, 278, 306	GC to AT	Flow sorting, sequencing	Neshat *et al* (1994)	p53 mutation occurs before aneuploidy
HGD	50% (29/58)	175, 248, 213, 245, 273	GC to AT	Flow sorting, sequencing	Prevo *et al* (1999)	More p53 mutation in aneuploid cells
Tumour	70% (14/20)	175, 248, 282	GC to AT	SSCP and sequencing	Gleeson *et al* (1998)	Five out of six cases same mutation in adjacent HGD, one case same mutation in adjacent LGD

Most studies have only identified p53 mutations in tumours and HGD tissue. However, a few reports suggest that p53 mutations are evident in LGD and metaplasia.

contains a list of published p53 mutation data for Barrett's tissues. Most of these data come from tumour samples, with some data available for adjacent premalignant Barrett's tissue (mostly dysplasia). The data in Table 1 are solely derived from molecular studies on p53 gene mutations in Barrett's tissue and do not include the inference of p53 mutations by immunohistochemistry (IHC). Using IHC as a surrogate for p53 mutation has been widely described as unsatisfactory (Hamelin *et al*, 1994; Schneider *et al*, 1996; Gleeson *et al*, 1998). From Table 1, it is evident that while some authors claim that p53 mutations are detectable in metaplastic tissue (Casson *et al*, 1991) or low-grade dysplastic tissue (Gleeson *et al*, 1998; Bian *et al*, 2001), most authors only detect mutations in tissues containing high-grade dysplasia or cancer (Hamelin *et al*, 1994; Audrezet *et al*, 1996). This discrepancy is probably because of the methodological shortcomings of these studies (Minamoto and Ronai, 2001). It is well known that there is an inverse correlation between the abundance of a genetic alteration (e.g. p53 mutation) and the ease with which it is detected (Minamoto and Ronai, 2001). In order to detect early p53 mutations in premalignant tissue of cancer-free patients, methodologies must enrich for mutated sequences at the expense of the more abundant nonmutated sequences (Minamoto and Ronai, 2001).

In this paper, we present a relatively simple molecular technique called restriction site mutation (RSM) (Jenkins *et al*, 1999,2001a,2001b) and assess its ability to detect early p53 mutations in premalignant Barrett's tissues from patients without clinically detectable adenocarcinoma. This approach has previously been employed in detecting p53 mutations in ulcerative colitis patients (Ambs *et al*, 1999) and gastritis patients (our laboratory, unpublished results). Restiction site mutation couples the sequence specificity of restriction enzymes to the amplification power of PCR in order to ‘fish out’ low-frequency mutated p53 sequences from among excess nonmutated sequences. The RSM technique exploits the fact that five of the eight main p53 mutation hotspots detected in cancers, that is, codons 175, 213, 248, 249, 282, contain restriction enzyme sites, thus allowing RSM to detect low-frequency mutations at these codons. In previous studies, we have shown that using spiked DNA samples, mutated hotspots are detectable even when in the presence of a 10000-fold excess of nonmutated sequences (Jenkins *et al*, 2001a,2001b).

The aim of this prospective study was to (i) assess whether RSM could detect early p53 mutations in premalignant Barrett's tissue, (ii) correlate the presence of any early p53 mutations with histological grade and (iii) assess the value of early p53 mutation as a biomarker for cancer risk in Barrett's patients.

## MATERIALS AND METHODS

### Collection of biopsies from Barrett's patients

Biopsies were obtained from consenting Barrett's patients during routine endoscopy clinics (ethical approval obtained). Biopsies for DNA analysis were obtained from Barrett's mucosa immediately adjacent to the biopsy sites for mucosa sent for histopathological analysis. These biopsies were obtained using large forceps and the whole biopsy (∼10 mg) was required for DNA extraction in order to provide sufficient DNA (∼30 *μ*g) for mutation analysis. Histopathological staging of the Barrett's patients was determined by the consensus view of a panel of three pathologists (APG, NT, VS). Barrett's patients were graded as dysplastic even if this was evident in only one of the biopsy specimens examined, as previously described (Prevo *et al*, 1999). A total of 38 patients with a range of histopathological stages of Barrett's oesophagus were recruited over a 12-month period (follow up: 9–20 months). Biopsies in four patients contained only squamous epithelium, 13 had Barrett's metaplasia without dysplasia, 14 had LGD and seven had HGD. In addition, paraffin-embedded archival tissue sections from surgically resected tissues were used to increase the numbers of HGD patients from seven to 11. From these 42 patients, 93 tissue samples were collected (average two biopsies per patient). The seven biopsies obtained from the four patients with squamous epithelium were considered as an internal control group in this study. Biopsies were immediately stored on ice until DNA extraction could be performed.

### DNA extraction

Biopsies were minced on a clean microscope slide using a clean scalpel blade. The minced tissues were then subjected to DNA extraction using a high-salt approach (Stratagene, Cambridge, UK). In the case of the paraffin-embedded tissue sections, the tissue was first scraped off five 5 *μ*m slides and incubated with 500 *μ*l of xylene to dissolve the paraffin. The tissue was subsequently pelleted by centrifugation and washed with 70% ethanol before proceeding with the DNA extraction (as above). After DNA extraction, the quantity and purity of the DNA was assessed by spectrophotometry at 260/280 nm and by 1% agarose electrophoresis. The DNA concentration was adjusted to 100 ng *μ*l^−1^ and the DNA stored at −20°C.

### Restriction site mutation analysis

The DNA extracted from oesophageal tissue samples was analysed for mutations occurring in five restriction enzyme sites of the human p53 gene. These restriction sites covered the hotspot codons 175, 213, 248, 249 and 282. A total of 465 RSM analyses were performed with the Barrett's samples (93 tissue samples analysed at each of five restriction enzyme sites). Table 2

**Table 2:** Details of the restriction enzymes employed in this study to detect p53 mutations at key tumour hotspot codons; also included are the PCR primer sequences for each hotspot codon and the amplification conditions

**p53 exon**	**Restriction enzymes**	**Digestion temperature** **(°C)**	**Forward primer**	**Reverse primer**	**PCR product size (bp)**	**Anneal temperature (°C)**	**Cycle number**
Exon 5							
Condon 175	*Hha* I GCGC	37	ccgcgccatggc	gcgctcatggtgg	75	60	31
			catct	ggg			
Exon 6							
Condon 213	*Taq* I TCGA	65	gtccccaggcct	taacccctcctccc	188	65	34
			ctgattcctc	agagaccccag			
Exon 7							
Condon 248	*Msp* I CCGG	37	ttggctctgactgt	agtgtgcagggtg	137	60	31
Condon 249	*Hae* III GGCC	37	accac	gcaag			
Exon 8							
Condon 282	*Msp* I CCGG	37	cctcttgcttctcttt	cttggtctcctccac	262	60	31
			tcctatcc	cgcttcttg			

contains details of the restriction enzymes used and the PCR conditions employed to amplify the mutant sequences. A previous study by ourselves has shown that using spiked tumour DNA bearing a p53 mutation, the RSM method can detect such mutations even when present among a 10000-fold excess of nonmutated DNA (Jenkins *et al*, 2001a,2001b).

#### Initial digestion

DNA (1.5 *μ*g containing 4 × 10^5^ copies of the p53 gene) was subject to restriction digestion with 10–20 U of the particular enzyme under test. In the case of the paraffin-embedded tissue sections, the low yield of DNA meant that only 500 ng of DNA was included in the RSM analysis. The initial digestion was performed in a volume of 20 *μ*l of magnesium-free *Taq* Polymerase buffer (Promega Corporation, Southampton, UK) supplemented with 1.5 mM MgCl_2_. The digestion was performed at the optimum temperature overnight (16 h). The following morning, a further 10 U of restriction enzyme was added for 1 h, to ensure maximum digestion of the nonmutated DNA.

#### Polymerase chain reaction amplification

Following digestion, the undigested (mutated) sequences were amplified by PCR using primers (Table 2) flanking the restriction site under examination (Cruachem, Glasgow, Scotland). The PCR amplification was performed in 1 × magnesium-free *Taq* polymerase buffer (Promega Corp.), along with 1.5 mM MgCl_2_, 100 *μ*M dNTPs (Promega Corp.), 10 pmol of each primer (Cruachem), 20 *μ*l of digested DNA and 2.5 U of *Taq* polymerase (Promega Corp.), in a final volume of 50 *μ*l. Positive (uncut DNA) and negative (no DNA) controls for the PCR step were also included.

#### Final digestion

After amplification, the PCR product (16 *μ*l) was further digested overnight in a final volume of 20 *μ*l, with 10 U of the restriction enzyme under test (this time in enzyme-specific digestion buffer) to remove any wild-type sequences that may have escaped the initial digestion.

#### Electrophoresis

**Figure 1:**
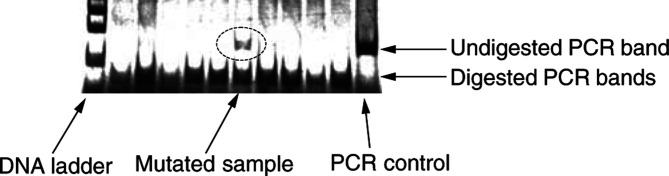
Example of an RSM experiment involving the *Msp* I restriction enzyme of codon 248 and DNA extracted from nine Barrett's tissues. The right-hand lane contains a positive control for the PCR step and shows the expected PCR band size. The left-hand lane contains a DNA ladder showing the 100, 200 and 300 bp bands. The central lanes contain DNA from Barrett's biopsies subject to RSM analysis. The highlighted lane contains an undigested band of the correct size and was subject to DNA sequencing where it was confirmed that it contained a GC to AT mutation. The remaining lanes (including the internal digestion control situated next to the PCR-positive control) have digested and hence do not contain mutations in this restriction site (codon 248).

The RSM products were then visualised on 5% polyacrylamide gels (Biorad, Hemel Hempstead, UK) and poststained with silver. Restriction enzyme-resistant RSM products were detected on the gels as undigested bands of the correct size, by comparison to the PCR-positive control (see Figure 1

).

As a control for inefficient enzyme digestion, all RSM experiments included commercially obtained human DNA (Promega Corp.) deemed to be free from mutations. Complete digestion of this control was a prerequisite for accepting mutations in accompanying samples. Fisher's exact test was employed to compare the frequency of p53 mutations between histologies.

### Sequencing

Mutations detected in this study by their resistance to restriction enzyme digestion were all confirmed by DNA sequencing. Enzyme-resistant products were reamplified by PCR using 1 *μ*l of the final RSM product (amplification for 10–12 cycles using appropriate thermal profiles). The reamplified samples were electrophoresed on 5% polyacrylamide gels and stained with silver to ensure that discrete bands were produced. These PCR products were then sequenced using a cycle sequencing kit (Beckman Coulter, High Wycombe, UK) and run on an automated DNA sequencer (CEQ2000 Beckman Coulter). Both strands were sequenced and only mutations apparent on both strands were accepted. In the case of the exon 5 mutated sequences, the PCR product (75 bp) was too small to be sequenced directly and was firstly cloned using the TA cloning method (Invitrogen NV Leek, The Netherlands).

## RESULTS

### Detection of p53 mutations in Barrett's tissues

No p53 mutations were detectable in any of the biopsies taken from the four patients with squamous epithelium, nor were any undigested products obtained with the internal digestion control. This suggests that mutation artefacts were not introduced during the methodology and that all mutations subsequently detected were genuinely present in the Barrett's tissues. Table 3

**Table 3:** p53 mutations detected in this study; details of the histology of the patients bearing p53 mutations are included, along with details of the mutation itself (base change, amino-acid change)

**Patient number**	**Histological stage**	**Mutations per biopsy**	**Mutated p53 condon**	**p53 mutation**	**Amino-acid change**
2	HGD	1/2	1 at 248	cgg-cag	Arg-Gln
3	HGD	2/4	2 at 248	cgg-cag	Arg-Gln
4	HGD	7/8	7 at 248	cgg-cag	Arg-Gln
6	BM	2/5	2 at 248	cgg-cag	Arg-Gln
8	HGD	2/5	2 at 248	cgg-cag	Arg-Gln
12	BM	1/2	1 at 175	cgc-cac	Arg-His
13	LGD	2/6	1 at 175	cgc-tgc	Arg-Gln
			1 at 248	cgg-cag	Arg-Cys
18	BM	1/1	1 at 248	cgg-cag	Arg-Gln
26	LGD	2/2	2 at 282	cgg-cgt	No change
31	LGD	1/2	1 at 248	cgg-cag	Arg-Gln
33	BM	1/2	1 at 248	cgg-tgg	Arg-Trp
38	LGD	3/2	2 at 248	cgg-cag	Arg-Gln
			1 at 175	cgc-cac	Arg-His
42	HGD	1/1	1 at 248	cgg-tgg	Arg-Trp

contains the details of the p53 mutations detected in Barrett's tissues. In total, 13 of the 38 patients with Barrett's oesophagus (36%) were shown to contain p53 mutations in at least one of their biopsies. Two patients (patients 13 and 38) possessed different p53 mutations in different biopsies, and seven out of 13 patients containing mutations were shown to contain a p53 mutation in more than one biopsy (with patient 4 having seven identical p53 mutations recovered from the eight biopsies available). The fact that seven out of eight different biopsies of patient 4 produced an identical p53 mutation may be considered a validation of the reproducibility of this approach in detecting p53 mutations in Barrett's tissues.

### Correlation of p53 mutation with histology

**Figure 2:**
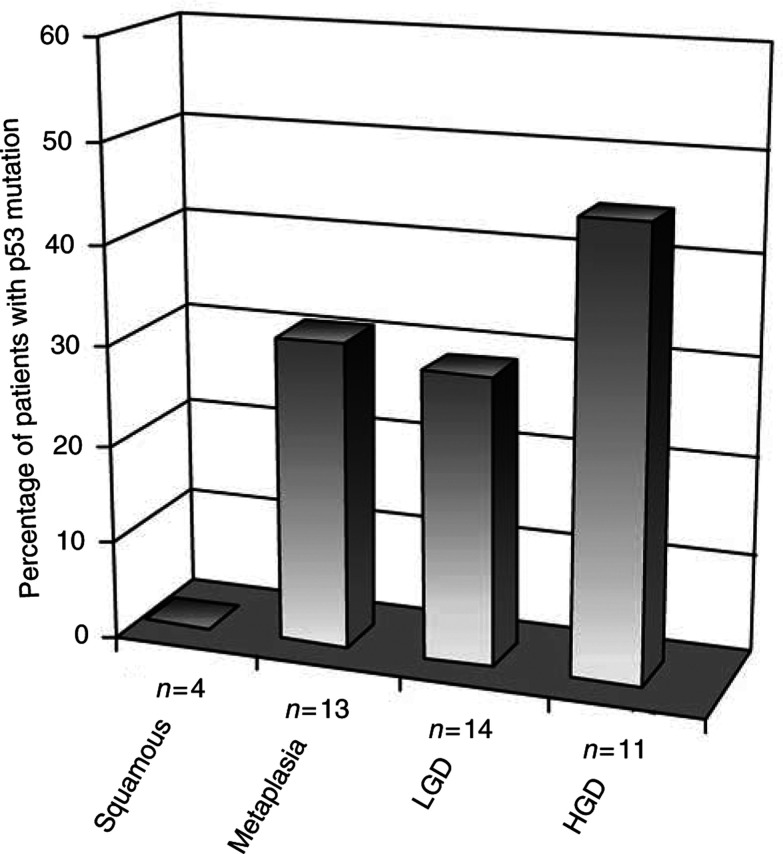
Role of p53 mutation in Barrett's progression. The proportion of patients with p53 mutations are correlated with histological grade. This figure shows the accumulation of p53 mutation with histological grade. From Table 1 it can be seen that 50 – 88% of adenocarcinomas have been shown to contain p53 mutations. Hence fitting well with the trend observed in this figure, however, the differing sensitivities of the methods used make true comparison difficult.

Of the 13 patients who were histologically classed as having metaplasia, four patients possessed a p53 mutation in at least one biopsy. In the case of LGD, four out of 14 patients possessed a p53 mutation. Of the 11 patients with HGD, a further five contained p53 mutations in a least one biopsy (see Figure 2

). There was no statistically significant difference in the number of p53 mutations in squamous tissue *vs* metaplastic tissue (*P*=0.4), or low-grade dysplastic tissue (*P*=0.33) or high-grade dysplastic tissue (*P*=0.15). The reported incidence of p53 gene mutation in adenocarcinomas is in the range 50 – 88% (Table 1), which corresponds well with the trend shown in Figure 2. However, it should be pointed out that we used a method here with a reasonably high sensitivity, whereas the previously published data were obtained with low-sensitivity methods, and hence comparisons may be difficult.

Seven of the 13 patients with p53 mutations were shown to contain multiple mutated tissue samples. As an indication of the clonal expansion of p53 mutated cells, six of these were dysplastic patients (three LGD, three HGD), suggesting that this clonal expansion step was linked to the histological progression of Barrett's tissues (*P*=0.13).

### p53 mutation types detected in Barrett's tissues

A total of 26 mutational events were detectable in the 465 RSM experiments performed. These mutations were mostly recovered from codon 248 (*Msp* I restriction site), with 21 of the mutations (80%) being found here, three mutations (12%) were recovered from codon 175 (*Hha* I restriction site), with the final two mutations (8%) at codon 282 (*Msp* I restriction site). The fact that the majority of p53 mutations were located at codon 248 suggests that this codon is particularly targeted in Barrett's progression. This is supported by the fact that codon 248 is the most common mutation hotspot in all human tumours (p53 mutation database: www.IARC.fr/p53/) and that Barrett's tumour p53 mutations, in particular, are often located at codon 248 (Table 1). No mutations were recovered from codons 213 (*Taq* I restriction site) or 249 (*Hae* III restriction site), despite reports of frequent polymorphisms in Barrett's patients at codon 213 (Hamelin *et al*, 1994; Campomenosi *et al*, 1996). Of the 26 mutations recovered, nearly all (92%) were GC to AT transitions at CpG sites, supporting previous data with regard to p53 mutations in Barrett's tumours (Gleeson *et al*, 1995,^1998^).

## DISCUSSION

Importantly, this study shows that a subset of tissues from nondysplastic Barrett's patients contain p53 mutations that may predispose those patients to tumour development. Previous reports have established that p53 mutation data can be used as a predictive marker for cancer development in Barrett's patients (Schneider *et al*, 1996), and that the presence of p53 mutations can predict poor 5-year survival rates of Barrett's tumour patients postoperatively (Schneider *et al*, 2000). p53 mutation has also been shown to be useful as a predictive marker for the occurrence of metastases in Barrett's tumour patients (Casson *et al*, 1991; Bian *et al*, 2001). Finally, p53 mutation data have also been used to predict the poor response of Barrett's tumour patients to photodynamic therapy (Krishnadath *et al*, 2000). p53 mutation therefore shows promise as a biomarker in Barrett's patients (Gleeson *et al*, 1998; Reid, 2001), but its use as an early biomarker for risk of cancer development relies on its detection as early as possible in premalignant Barrett's tissues.

We have shown here that the application of a molecular technique, that is, RSM, has allowed the detection of low-frequency p53 mutations in premalignant Barrett's tissues. As has been pointed out previously, it is not feasible to use direct DNA sequencing as a clinical tool to detect p53 mutation in Barrett's patients (Reid, 2001). However, the RSM method appears to be well suited in scanning the hotspot codons of p53 for early mutations, which would be undetectable by less-sensitive contemporary techniques. We show that the appearance of p53 mutated cells can occur at the metaplasia stage, with the clonal expansion of these mutated cells occurring at the dysplastic stage.

It is interesting to note that two of the patients were shown to have different p53 mutations in different biopsies, thus indicating that divergent clonal evolution may be occurring in their Barrett's segments. It has previously been suggested that early neoplasia may well have a multifocal nature leading to oligoclonal development (Jankowski *et al*, 1999). These lines of evidence point to the Barrett's epithelium being the subject of high levels of genetic evolution as a result of continuous exposure to potent mutagenic agents, for example, ROS. Most of the p53 mutations detected were present at CpG sites. CpG sites are known to be susceptible to spontaneous deamination of methylated cytosine, leading to the conversion of C to T (Pfeiffer, 2000). This process is thought to be enhanced by exposure to ROS (Ambs *et al*, 1999), possibly implicating inflammation-induced ROS in the development of tumours in Barrett's patients.

There are indeed notable similarities in the mutation profile (types and positions) detected here in Barrett's patients and those p53 mutations previously shown by ourselves to be induced by the model ROS hydrogen peroxide *in vitro* (Jenkins *et al*, 2001a,2001b). This adds weight to the argument that ROS produced during inflammation may play a role in cancer development in Barrett's tissues and may be responsible for the p53 mutations detected here.

The potential value of p53 as a biomarker depends upon whether it can be shown that possession of a p53 mutation either affects the risk of cancer progression in Barrett's patients, or affects the rate at which this sequence unfolds. Only long-term follow-up of these patients will answer this question. The fact that p53 mutation abundance appears to increase during histological progression suggests that this abnormality plays an important driving role in carcinogenesis in Barrett's tissues.
